# Reporting of Factorial Randomized Trials Extension of the CONSORT 2010 Statement

**DOI:** 10.1001/jama.2023.19793

**Published:** 2023-12-05

**Authors:** Brennan C. Kahan, Sophie S. Hall, Elaine M. Beller, Megan Birchenall, An-Wen Chan, Diana Elbourne, Paul Little, John Fletcher, Robert M. Golub, Beatriz Goulao, Sally Hopewell, Nazrul Islam, Merrick Zwarenstein, Edmund Juszczak, Alan A. Montgomery

**Affiliations:** MRC Clinical Trials Unit at UCL, London, United Kingdom; Nottingham Clinical Trials Unit, School of Medicine, University of Nottingham, Nottingham, United Kingdom; Institute for Evidence-Based Healthcare, Bond University, Queensland, Australia; Nottingham Clinical Trials Unit, School of Medicine, University of Nottingham, Nottingham, United Kingdom; Women’s College Research Institute, University of Toronto, Toronto, Ontario, Canada; London School of Hygiene and Tropical Medicine, London, United Kingdom; Primary Care Research Centre, School of Primary Care, Population Sciences and Medical Education, Faculty of Medicine, University of Southampton, Southampton, United Kingdom; The BMJ, BMA House, Tavistock Square, London, United Kingdom; Department of Medicine, Northwestern University Feinberg School of Medicine, Chicago, Illinois; Health Services Research Unit, University of Aberdeen, Aberdeen, Scotland; Oxford Clinical Trials Research Unit, University of Oxford, Oxford, United Kingdom; Primary Care Research Centre, School of Primary Care, Population Sciences and Medical Education, Faculty of Medicine, University of Southampton, Southampton, United Kingdom; The BMJ, BMA House, Tavistock Square, London, United Kingdom; Centre For Studies in Family Medicine, Schulich School of Medicine and Dentistry, Western University, London, Ontario, Canada; Nottingham Clinical Trials Unit, School of Medicine, University of Nottingham, Nottingham, United Kingdom

## Abstract

**Importance:**

Transparent reporting of randomized trials is essential to facilitate critical appraisal and interpretation of results. Factorial trials, in which 2 or more interventions are assessed in the same set of participants, have unique methodological considerations. However, reporting of factorial trials is suboptimal.

**Objective:**

To develop a consensus-based extension to the Consolidated Standards of Reporting Trials (CONSORT) 2010 Statement for factorial trials.

**Design:**

Using the Enhancing the Quality and Transparency of Health Research (EQUATOR) methodological framework, the CONSORT extension for factorial trials was developed by (1) generating a list of reporting recommendations for factorial trials using a scoping review of methodological articles identified using a MEDLINE search (from inception to May 2019) and supplemented with relevant articles from the personal collections of the authors; (2) a 3-round Delphi survey between January and June 2022 to identify additional items and assess the importance of each item, completed by 104 panelists from 14 countries; and (3) a hybrid consensus meeting attended by 15 panelists to finalize the selection and wording of items for the checklist.

**Findings:**

This CONSORT extension for factorial trials modifies 16 of the 37 items in the CONSORT 2010 checklist and adds 1 new item. The rationale for the importance of each item is provided. Key recommendations are (1) the reason for using a factorial design should be reported, including whether an interaction is hypothesized, (2) the treatment groups that form the main comparisons should be clearly identified, and (3) for each main comparison, the estimated interaction effect and its precision should be reported.

**Conclusions and Relevance:**

This extension of the CONSORT 2010 Statement provides guidance on the reporting of factorial randomized trials and should facilitate greater understanding of and transparency in their reporting.

In a factorial trial, 2 or more interventions are assessed in a single study by randomizing participants to multiple factors.^1–14^ In a 2 × 2 trial with factors A and B, participants are randomized to receive intervention A or its comparator and also to intervention B or its comparator, meaning participants are assigned to 1 of 4 treatment groups: A alone, B alone, A plus B, or neither A nor B (Table 1).

Factorial designs are used to address different research questions (ie, estimands; Box 1). They can be used to evaluate more than 1 intervention in a single trial without increasing the sample size (“2-in-1” trials), to evaluate whether interventions interact, or to identify the best combination of interventions.^8,13,15,16^ These disparate aims require different methodology, including sample size calculations and analysis strategies. Factorial trials also have additional methodological complexities compared with other trial designs, including choice of what treatment groups to include in main comparisons, how potential interactions should be handled during analysis, and nonconcurrent enrollment of participants.^1,2,4,6,10–13,17^

An extension of the Consolidated Standards of Reporting Trials (CONSORT) 2010 checklist for the reporting of factorial trials is presented in this article.^18,19^ A glossary of key terms is provided in Box 1.

## Methods

This CONSORT extension development occurred in parallel with the Standard Protocol Items: Recommendations for Interventional Trials (SPIRIT) extension for factorial trials.^20^ First, we performed a scoping review using a MEDLINE search from inception to May 2019 to create an initial list of reporting recommendations applicable to factorial trials. Second, we performed a 3-round Delphi survey (January to June 2022; 104 panelists from 14 countries) to identify additional items and assess the importance of each item. Third, an expert consensus meeting (September 6-7, 2022; 15 panelists) was held to establish the final checklist. Item wording was finalized after the meeting through iterative discussions.

## Results

The checklist for the reporting of factorial randomized trials includes 16 modified items and 1 new item (Table 2). Reporting items for abstracts of factorial randomized trials are provided in Table 3.^21,22^

The scoping review identified 31 recommendations pertinent to reporting factorial trials, which were evaluated in the Delphi survey. Thirty-two recommendations met the criteria to be evaluated at the consensus meeting (1 recommendation was added in round 2 of the Delphi survey).

Given the variation in terminology used to describe factorial trials, items in this statement have been written to replace the original CONSORT items. Users are advised to refer to definitions of key terms in Box 1. This article contains brief explanations of the modified items in the CONSORT factorial extension. Details for interpretation of each item, and examples of good reporting, will be presented in a separate “explanation and elaboration” article.

### CONSORT Checklist Extension for Factorial Randomized Trials

#### Item 1a. CONSORT 2010: Identification as a randomized trial in the title

##### Extension for factorial trials: Identification as a factorial randomized trial in the title

Notifying readers of the factorial design alerts them to potential implications of the design for analysis and interpretation.^2,4,5,8,23,24^

#### Item 2a. CONSORT 2010: Scientific background and explanation of rationale

##### Extension for factorial trials: Rationale for using a factorial design, including whether an interaction is hypothesized

Different research hypotheses require different methodology. By clarifying the rationale for using the factorial design, as well as whether an interaction is hypothesized, readers are signposted toward the key objectives and alerted to the assumptions and methodological features required.^1,4–6,24^

#### Item 2b. CONSORT 2010: Specific objectives or hypotheses

##### Extension for factorial trials: A statement of which treatment groups form the main comparisons

In factorial trials, interventions can be compared in different ways. In a 2 × 2 factorial trial with factors A and B, the treatment effect for intervention A vs its comparator can be estimated by comparing (1) participants randomized to receive A vs not A; (2) those randomized to receive A alone vs neither A nor B; or (3) those randomized to receive A plus B vs B alone. These alternative comparisons can target different estimands and are underpinned by different assumptions (Box 2).^4,6,11^ An estimand describes the target treatment effect to be estimated from the trial.

#### Item 3a. CONSORT 2010: Description of trial design (such as parallel, factorial) including randomization ratio

##### Extension for factorial trials: Description of the type of factorial trial (such as a full or partial, number of factors, and levels within each factor)

Most factorial trials use a “full” factorial design, whereby all participants are eligible to be randomized to all combinations of factors and factor levels.^9,25,26^ Other designs include “fractional” factorial designs (where some combinations of factors are omitted) and “partial” factorial designs (where some participants are only eligible to be randomized to certain factors), which require alternative methodology.^1,27^

#### Item 4a. CONSORT 2010: Eligibility criteria for participants

##### Extension for factorial trials: Eligibility criteria for each factor, noting any differences, if applicable

Differences in eligibility criteria across factors can have implications for the design and analysis and can increase the risk of bias if not handled properly. For instance, participants who are not eligible for randomization to a specific factor should not be included in the comparison for that factor, because their inclusion means the analysis is no longer based on a randomized comparison, which can lead to confounding bias.^1,27^

#### Item 7a. CONSORT 2010: How sample size was determined

##### Extension for factorial trials: How sample size was determined for each main comparison, including whether an interaction was assumed in the calculation

Sample size calculations for factorial designs are more complicated than in standard parallel-group designs. In some factorial trials, the planned main comparisons may require different sample sizes if they are expected to produce different effect sizes or if the choice of primary outcome varies for each factor.^6,28^ If an interaction is hypothesized, the sample size may need to be increased.^1,2,6,24^

Box 1 **Glossary of Terms Used in the Extension of the CONSORT 2010 Statement**

Comparison: What treatment groups will be compared against each other. For example, the effect of intervention A may be estimated by comparing all participants randomized to active A (treatment groups active A plus high-dose B and active A plus low-dose B) with all participants randomized to placebo A (treatment groups placebo A plus high-dose B and placebo A plus low-dose B).

Estimand: A description of the treatment effect to be estimated from the trial, including specification of the treatment conditions, population, end point, summary measure, and strategies to handle intercurrent events. Factorial trials should additionally specify how the other factor(s) are to be handled in the estimand (eg, whether interest lies in the effect of active A plus low-dose B vs placebo A plus low-dose B or else active A plus high-dose B vs placebo A plus high-dose B).

Factor: Each intervention and its comparator(s) together comprise a factor (eg, active A and placebo A together comprise one factor and high-dose B and low-dose B together make up the other factor).

Factorial analysis: Also called an “at-the-margins” analysis. All participants randomized to active A (treatment groups active A plus high-dose B and active A plus low-dose B) are compared against all those randomized to placebo A (placebo A plus high-dose B and placebo A plus low-dose B) and similarly for the factor B comparison.

Factorial trial: When 2 or more interventions are assessed in the same participants within a single study.

Fractional factorial design: Some combinations of factors are omitted. For example, in a trial with 3 factors (A, B, and C), participants may be randomized to 4 of the 8 possible combinations.

Full factorial design: All factors and levels are combined so the design comprises all possible combinations of factor levels and all participants are eligible to be randomized for each factor.

Interaction: Interactions occur when the effect of one treatment depends on whether participants also receive the other treatment (eg, active A may be less effective when used alongside high-dose B than when used with low-dose B). Interactions may occur for biological or social reasons (eg, if receipt of one treatment affects the mechanism of action for the other). Interactions may also occur due to choice of analysis scale (eg, active A may be equally effective with high-dose B as with low-dose B when measured on the risk ratio scale, but less effective on the risk difference scale). Trials interested in evaluating whether treatments interact are typically interested in biological/social interactions, while trials that use analyses that require an assumption of no interaction are affected by any type of interaction.

Level within factors: The specific interventions within a factor are the levels (eg, active A and placebo A are the 2 levels of factor A).

Main comparison(s): The comparison(s) that will primarily be used to draw conclusions about effectiveness of each intervention.

Multiarm analysis: Also called an “inside-the-table” analysis. The treatment groups active A plus low-dose B, placebo A plus high-dose B, and active A plus high-dose B are each compared against placebo A plus low-dose B (double-control).

Treatment group: The unique combinations of factors and levels to which participants can be randomized (eg, active A plus high-dose B comprise one treatment group and active A plus low-dose B another).

Partial factorial design: Some participants are not randomized to certain factors. For example, a subset of participants will only be randomized between active A vs control A and will receive control B automatically.

#### Item 7b. CONSORT 2010: When applicable, explanation of any interim analyses and stopping guidelines

##### Extension for factorial trials: When applicable, explanation of any interim analyses and stopping guidelines, noting any differences across main comparisons and reasons for differences

The plan for interim analyses and subsequent stopping guidelines may be different for each factor.^27^ If one factor is stopped before the other, there may be implications for randomization, choice of comparator, or analysis.^1,27,29^

#### Item 8b. CONSORT 2010: Type of randomization; details of any restriction (such as blocking and block size)

##### Extension for factorial trials: If applicable, whether participants were randomized to factors at different points

Participants may be randomized to factors at different time points (eg, for factor A at diagnosis of disease then for factor B after treatment A is complete). The time point of randomization for each factor may inform key design features, such as the baseline period, duration of follow-up, and likelihood of treatments interacting.^2^

#### Item 12a. CONSORT 2010: Statistical methods used to compare groups for primary and secondary outcomes

Extension for factorial trials: Statistical methods used for each main comparison for primary and secondary outcomes, including:

- **Whether the target treatment effect for each main comparison pertains to the effect in the presence or absence of other factors** The statistical methods alone are not always sufficient to allow readers to understand the exact treatment effect (estimand) being estimated.^30–32^ In factorial trials, the treatment groups used for comparison are not always the same as those in which there is interest in estimating the treatment effect.^11,33^ For example, many factorial trials use a factorial analysis to compare “all A” vs “all not A” for reasons of efficiency, even though interest really lies in the effect of A alone vs control (the effect of A in the absence of B) or, alternatively, the effect of A plus B vs B alone (the effect of A in the presence of B) if treatment B has been demonstrated to be effective.^11^ A clear description of the target treatment effect, including whether it pertains to the effect in the presence or absence of other factors, allows readers to understand the exact question being addressed.^11,30,31,34^
- **Approach to analysis, such as factorial or multiarm** Different statistical methods can be used to analyze a factorial trial depending on the estimand of interest. In a factorial (or “at-the-margins”) analysis, all participants randomized to factor A (A alone and A plus B) are compared with all those not randomized to A (B alone and double-control).^2,4,6,11,35,36^ Alternatively, in a multiarm (or “inside-the-table”) analysis, the trial is analyzed as if a multiarm design had been used.^2,4–6,10–12,17,23,35,36^ The 2 approaches offer different benefits and require different assumptions (Box 2).
- **How the approach was chosen, such as prespecified or based on estimated interaction**
  Using a test of interaction to guide the choice of analysis can introduce bias and is not recommended.^17^ Clarification of whether the final analysis approach was prespecified based on prior knowledge or an assumption of no interaction or chosen based on the size of the estimated interaction helps alert readers to any risk of bias associated with the analysis approach.
- **Method(s) used to evaluate statistical interaction(s)**
  It is recommended practice to evaluate the presence of statistical interactions, either because analyses rely on the assumption that treatments do not interact or because the interaction is itself of direct interest.^2,4–6,10,11,24^ The presence of an interaction may depend on the scale of analysis (eg, an interaction may be present on the risk difference scale, but not the risk ratio scale), so careful consideration should be given to the choice of scale. Reporting details of how interaction(s) were evaluated, and on what scale, enables readers to understand the appropriateness of method(s).
- **If factorial approach used, whether factors were adjusted for each other**
  Factorial analyses can be adjusted for whether participants were randomized to the other factor(s) by including a term for this in the statistical model.^2,6,11,28^ This can increase statistical power and, in some cases, failure to adjust for the other factors can introduce bias for certain estimands.^11^
- **If applicable, how nonconcurrent recruitment to factors was handled**
  Nonconcurrent recruitment, in which certain participants are not randomized for some factors (eg, if the trial used a partial factorial design or recruitment to one factor is paused or terminated), can induce bias if not handled correctly during analysis (see item 4a).^1,27^

#### Item 13a. CONSORT 2010: For each group, the numbers of participants who were randomly assigned, received intended treatment, and were analyzed for the primary outcome

##### Extension for factorial trials: For each main comparison, the number of participants who were randomly assigned, received intended treatment, and were analyzed for the primary outcome

For factorial trials, especially those beyond a 2 × 2 design, it can be difficult for readers to identify the relevant participant flow because this information may differ across main comparisons. Presenting this information for each main comparison increases clarity and understanding.^2,4–6,8,10,35^

#### Item 14a. CONSORT 2010: Dates defining the periods of recruitment and follow-up

##### Extension for factorial trials: Dates defining the periods of recruitment and follow-up for each factor, noting any differences with reasons

If periods of recruitment are different across factors, participants enrolled after one factor has stopped recruitment will only be eligible to be randomized for the ongoing factor(s), posing similar statistical issues as in a partial factorial design (see CONSORT item 4a).^27^

Box 2 **Estimands for Factorial Trials**

**Estimands for factorial trials**

An estimand describes a research question a trial sets out to address (Box 1).

Different types of estimands may be specified for factorial trials depending on the aims.

An estimand for the effect of treatment A could be defined based on a comparison of treatment A vs not A if no one received treatment B or as the effect of A vs not A if everyone received treatment B.

The former may be more common for “2-in-1” factorial trials because it provides the effect of treatment A that would be seen in a parallel-group design where treatment B is not used. However, either estimand may be of interest.

Alternatively, an estimand for treatment A could also be defined based on the effect of A vs not A averaged across those who do and those who do not receive treatment B.^a^ Because this estimand does not typically reflect how treatments are used in practice, other choices are usually more relevant for 2-in-1 trials.

For trials in which the aim is to determine whether treatments interact, the estimand may be based around the difference between the effect of treatment A if no one received treatment B vs the effect if everyone received treatment B.

**Implications for statistical analysis^b^**

The method of statistical analysis should be determined by the estimand (ie, research question).

Two-in-1 trials typically use a factorial analysis because this realizes the efficiency gains inherent to the factorial design. However, because this analysis averages across the 2 strata of those randomized to receive and not receive B, it only estimates the “effect of treatment A if no one receives B” if treatments A and B do not interact. When treatments do interact, it estimates the mean effect of A across the strata of B. Therefore, assessment of the interaction is essential to determine whether the factorial analysis is estimating the desired estimand.

A multiarm (“inside-the-table”) analysis could also be used to estimate the effect of treatment A if no one receives B, and is unbiased regardless of whether treatments A and B interact. However, it does not realize the efficiency gained through using a factorial design, so it is less frequently used for 2-in-1 trials.

^a^This averaging could correspond to the study proportions randomized to receive treatment B and not B or to some other proportions defined by the investigators. The exact method of determining the mean therefore needs to be made explicit.

^b^A factorial analysis can be used to estimate either (1) the effect of A if no one received B; (2) the effect of A if everyone received B; or (3) the effect of A averaged over those who received and did not receive B according to the study proportions. The first 2 of these estimates require the assumption of no interaction, but the analysis for the third does not. A multiarm analysis can be used to estimate by either comparing A alone vs double-control (as described above) or comparing A plus B vs B alone. These do not require the assumption of no interaction. If interest lies in the effect of A averaged over those who do and do not receive B according to proportions other than the study proportions, this could be estimated by first estimating the effect of A separately in both stratum (those who receive and do not receive B) then taking a weighted average of these according to the desired proportions. This analysis does not require the assumption of no interaction.^11^

#### Item 17a. CONSORT 2010: For each primary and secondary outcome, results for each group and the estimated effect size and its precision (such as 95% CI)

Extension for factorial trials: For each primary and secondary outcome, results for each main comparison, the estimated effect size, and its precision (such as 95% CI)

For each primary outcome, the estimated interaction effect and its precision

##### If done, estimated interaction effects and precision for secondary outcomes

For factorial trials predicated on the assumption of no interaction (2-in-1 trials) or those in which the interaction is of main interest, evaluation of interactions is essential to interpretation.^2,4–6,10,11,24^ The size of the estimated interaction effect should be presented along with a measure of precision, such as the 95% CI.^2,5,6^ For trials in which evaluation of interaction(s) is not deemed essential, this decision should be justified.

#### Item 18b. CONSORT 2010: New item (additional data summaries)

##### New item for factorial trials: Participant flow, losses and exclusions, and outcome data (including primary and secondary outcomes, harms, and adherence) presented by treatment groups

Outcomes and other postrandomization data such as adherence, harms, and participant flow may be affected when treatments interact.^26^ Presentation of such data by treatment group (eg, groups A alone, B alone, A plus B, and double-control in a 2 × 2 trial), in addition to presentation by main comparisons, allows readers to assess to what extent such data may be unduly influenced by interactions due to the factorial design.^3–6,8,10^

## Discussion

This extension to the CONSORT 2010 Statement provides guidance for reporting factorial trials. The extension checklist represents the minimum essential requirements for reporting of factorial trials; for some trials there will be additional items that are important to report. For instance, if primary or secondary outcomes differ by factor, this should be reported. Similarly, if multiple testing is deemed to be an issue, authors should report how this was handled.

This extension was developed in conjunction with the SPIRIT extension for factorial trials. Together, these guidelines provide a framework for cohesive reporting from the trial protocol to publication of results. The latest version of this and other CONSORT statements can be found online (https://www.equator-network.org/).

### Limitations

This study has several limitations. First, this extension was developed for studies in which results for each factor would be published simultaneously in the same article. This may not always be feasible, for instance, due to the early stopping of one factor or because each factor requires different durations of follow-up. In this case, we recommend that each publication follows the checklist as far as possible, while recognizing that the information for some items might differ. For example, each article could report how the sample size was determined for the relevant comparison, rather than the sample size calculations for each comparison (although each calculation would need to clarify whether an interaction was assumed).

Second, although the EQUATOR guidelines were followed to develop this guideline, Delphi respondents were self-selecting and consensus meeting panelists were purposively identified based on their expertise. Therefore, although results represent the views of a large, multinational group of experts and end users, the views of individuals not well represented by the Delphi survey or consensus meeting panelists may differ. However, the systematic and evidence-based approach used to develop this guideline, including a rigorous scoping review, should help mitigate the potential effects of these limitations.

## Conclusion

This extension of the CONSORT 2010 Statement provides specific guidance for the reporting of factorial randomized trials to facilitate greater transparency and completeness in the reporting of these trials.

## Supplementary Material

Supplement

figure-Fig 1

figure-Fig 2

figure-Fig 3

## Figures and Tables

**Table 1:** Example of a 2 × 2 Factorial Randomized Trial

	Treatment B (high-dose)^a,b^	Treatment B (low-dose)^a,b^
**Treatment A** **(active)** ^a,b^	Active A + high-dose B^c^	Active A + low-dose B^c^
**Treatment A** **(placebo)** ^a,b^	Placebo A + high-dose B^c^	Placebo A + low-dose B^c^

a A and B are factors.

b Active A and placebo A are levels within factor A and high-dose B and low-dose B are levels within factor B. Low-dose B is taken as the control condition for factor B.

c Active A plus high-dose B, active A plus low-dose B, placebo A plus high-dose B, and placebo A plus low-dose B are the treatment groups. In a “full” factorial trial all participants are eligible to be randomized between each of the 4 treatment groups; in a “partial” factorial trial, a subset of participants would only be randomized between high- and low-dose B and assigned to placebo A without randomization. In a “factorial” analysis, all participants allocated to intervention A (active A plus low-dose B and active A plus high-dose B) are compared against those not allocated to A (placebo A plus low-dose B and placebo A plus high-dose B), and similarly for the comparison for intervention B. In a “multiarm” analysis, each of the treatment groups are compared against control (eg, active A plus high-dose B, active A plus low-dose B, and placebo A plus high-dose B are all compared against placebo A plus low-dose B).

**Table 2:** CONSORT Checklist of Information to Include When Reporting Factorial Randomized Trials^a,b^

Section			Item No.	CONSORT 2010 Statement checklist item	Extension for factorial trials
Title and abstract						
	Title		1a	Identification as a randomized trial in the title	Identification as a factorial randomized trial in the title
	Abstract		1b	Structured summary of trial design, methods, results, and conclusions (for specific guidance see CONSORT for abstracts)	See separate factorial checklist for abstracts
Introduction						
	Background		2a	Scientific background and explanation of rationale	Scientific background and rationale for using a factorial design, including whether an interaction is hypothesized
	Objectives		2b	Specific objectives or hypotheses	Specific objectives or hypotheses and a statement of which treatment groups form the main comparisons^b^
Methods						
	Trial design		3a	Description of trial design (such as parallel, factorial) including allocation ratio	Description of the type of factorial trial (such as full or partial, number of factors, levels within each factor^b^) and allocation ratio
	Change from protocol		3b	Important changes to methods after trial commencement (such as eligibility criteria), with reasons	
	Participants		4a	Eligibility criteria for participants	Eligibility criteria for each factor, noting any differences, if applicable
	Setting and location		4b	Settings and locations where the data were collected	
	Interventions		5	The interventions for each group with sufficient details to allow replication, including how and when they were actually administered	
	Outcomes		6a	Completely defined pre-specified primary and secondary outcome measures, including how and when they were assessed	
	Changes to outcomes		6b	Any changes to trial outcomes after the trial commenced, with reasons	
	Sample size		7a	How sample size was determined	How sample size was determined for each main comparison, including whether an interaction was assumed in the calculation
	Interim analyses and stopping guidelines		7b	When applicable, explanation of any interim analyses and stopping guidelines	When applicable, explanation of any interim analyses and stopping guidelines, noting any differences across main comparisons and reasons for differences
	Randomization					
		Sequence generation	8a	Method used to generate the random allocation sequence	
		Sequence generation	8b	Type of randomization; details of any restriction (such as blocking and block size)	Type of randomization, details of any restriction (such as blocking and block size), and, if applicable, whether participants were randomized to factors at different time points
	Allocation concealment mechanism		9	Mechanism used to implement the random allocation sequence (such as sequentially numbered containers), describing any steps taken to conceal the sequence until interventions were assigned	
	Implementation		10	Who generated the random allocation sequence, who enrolled participants, and who assigned participants to interventions	
	Blinding		11a	If done, who was blinded after assignment to interventions (for example, participants, care providers, those assessing outcomes)	
	Similarity of interventions		11b	If relevant, description of the similarity of interventions	
	Statistical methods		12a	Statistical methods used to compare groups for primary and secondary outcomes	Statistical methods used for each main comparison for primary and secondary outcomes, including: Whether the target treatment effect for each main comparison pertains to the effect in the presence or absence of other factors Approach to analysis, such as factorial or multiarm How the approach was chosen, such as prespecified or based on estimated interaction If factorial approach was used, whether factors were adjusted for each other If applicable, how nonconcurrent recruitment to factors was handled Method(s) used to evaluate statistical interaction(s)
	Additional analyses		12b	Methods for additional analyses, such as subgroup analyses and adjusted analyses	
Results						
	Participant flow (a diagram is strongly recommended)		13a	For each group, the numbers of participants who were randomly assigned, received intended treatment, and were analyzed for the primary outcome	For each main comparison, the number of participants who were randomly assigned, received intended treatment, and were analyzed for the primary outcome
	Losses and exclusions		13b	For each group, losses and exclusions after randomization, together with reasons	For each main comparison, losses and exclusions after randomization, together with reasons
	Recruitment		14a	Dates defining the periods of recruitment and follow-up	Dates defining the periods of recruitment and follow-up for each factor, noting any differences, with reasons
	Trial end		14b	Why the trial ended or was stopped	
	Baseline data		15	A table showing baseline demographic and clinical characteristics for each group	A table showing baseline demographic and clinical characteristics for each main comparison
	Numbers analyzed		16	For each group, the number of participants (denominator) included in each analysis and whether the analysis was by original assigned groups	For each main comparison, the number of participants (denominator) included in each analysis and whether the analysis was by original assigned groups
	Outcomes and estimation		17a	For each primary and secondary outcome, results for each group and the estimated effect size and its precision (such as 95% CI)	For each primary and secondary outcome, results for each main comparison, the estimated effect size, and its precision (such as 95% CI) For each primary outcome, the estimated interaction effect and its precision If done, the estimated interaction effects and precision for secondary outcomes
	Binary outcomes		17b	For binary outcomes, presentation of both absolute and relative effect sizes is recommended	
	Ancillary analyses		18a	Results of any other analyses performed, including subgroup analyses and adjusted analyses, distinguishing prespecified from exploratory	
	Additional data summaries^c^		18b		Participant flow, losses and exclusions, baseline data, and outcome data (including primary and secondary outcomes, harms, and adherence) presented by treatment groups^b^
	Harms		19	All important harms or unintended effects in each group (for specific guidance see CONSORT for harms)	All important harms or unintended effects for each main comparison
Discussion						
	Limitations		20	Trial limitations, addressing sources of potential bias, imprecision, and, if relevant, multiplicity of analyses	
	Generalizability		21	Generalizability (external validity, applicability) of the trial findings	
	Interpretation		22	Interpretation consistent with results, balancing benefits and harms, and considering other relevant evidence	
Other information						
	Registration		23	Registration number and name of trial registry	
	Protocol		24	Where the full trial protocol can be accessed, if available	
	Funding		25	Sources of funding and other support (such as supply of drugs), role of funders	

a It is strongly recommended that this checklist is read in conjunction with the CONSORT 2010 checklist (https://www.equator-network.org/reporting-guidelines/consort/) and Statement Explanation and Elaboration paper^18^ for important clarification on the items. The CONSORT-factorial Checklist is licensed by the CONSORT-factorial Group under the Creative Commons Attribution-NonCommercial-NoDerivs 4.0 International license.

b Each overall intervention group to be compared is a *factor* (eg, in a 2 × 2 trial with factors A and B, active A and control A together comprise one factor and active B and control B together comprise another factor). The specific interventions within a factor are the *levels* (eg, active A and control A are the 2 levels of factor A). The unique combinations of factors and levels are *treatment groups* (eg, in a 2 × 2 trial with factors A and B there will be 4 treatment groups: active A plus control B, active A plus active B, etc). What treatment groups will be compared against each other to draw main conclusions about the effectiveness of each intervention is the *main comparison*.

c New item.

**Table 3:** Items to Include When Reporting a Randomized Factorial Trial in a Journal or Conference Abstract^a^

Item		CONSORT for abstracts checklist item	Extension for factorial trials
Title		Identification of the study as randomized	Identification of the study as a factorial randomized trial
Authors^b^		Contact details for the corresponding author	
Trial design		Description of the trial design (eg, parallel, cluster, noninferiority)	Description of the trial design (eg, parallel, cluster, noninferiority) and number of factors (eg, 2 × 2)
Methods			
	Participants	Eligibility criteria for participants and the settings where the data were collected	Eligibility criteria for each factor, noting any differences if applicable, and the settings where the data were collected
	Interventions	Interventions intended for each group	
	Objective	Specific objective or hypothesis	
	Outcome	Clearly defined primary outcome for this report	
	Randomization	How participants were randomized to interventions	
	Blinding (masking)	Whether or not participants, caregivers, and those assessing the outcomes were blinded to group assignment	
Results			
	Numbers randomized	Number of participants randomized to each group	Number of participants randomized for each main comparison
	Recruitment	Trial status	
	Numbers analyzed	Number of participants analyzed in each group	Number of participants analyzed for each main comparison
	Outcome	For the primary outcome, a result for each group and the estimated effect size and its precision	For the primary outcome, results for each main comparison, the estimated effect size and its precision, and estimated interaction effect and its precision
	Harms	Important adverse events	Important adverse events for each main comparison
Conclusions		General interpretation of the results	
Trial registration		Registration number and name of trial register	
Funding		Source of funding	

a The CONSORT-factorial Abstract Checklist is licensed by the CONSORT-factorial Group under the Creative Commons Attribution-NonCommercial-NoDerivs 4.0 International license.

b This item is specific to conference abstracts.

## Data Availability

See the Supplement.
