# Supplementary material for: Reporting of Factorial Randomized Trials Extension of the CONSORT 2010 Statement
Source: JAMA. Author manuscript; Available in PMC 2025 Jan 25. (PMC7617336; doi:10.1001/jama.2023.19793)
Supplement: Supplement [file EMS202149-supplement-Supplement.pdf]

Data Sharing Statement

Data
Data available: No

Additional Information
Explanation for why data not available: Data can be requested by contacting the corresponding author.
