# Supplementary material for: Reporting of Factorial Randomized Trials Extension of the CONSORT 2010 Statement
Source: JAMA. Author manuscript; Available in PMC 2025 Jan 25. (PMC7617336; doi:10.1001/jama.2023.19793)
Supplement: figure-Fig 1 [file EMS202149-supplement-figure-Fig_1.ppt]

## Slide 1
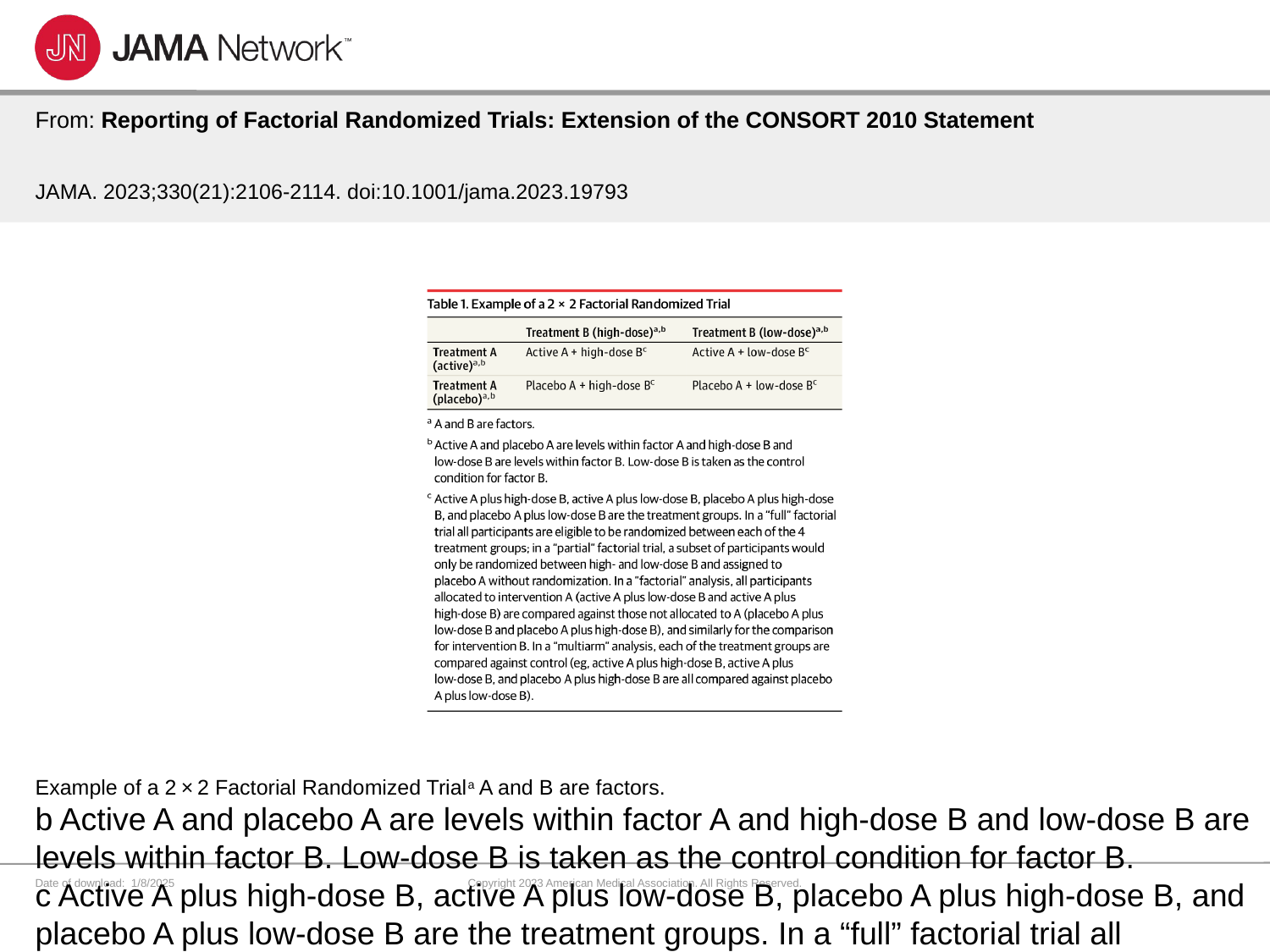

From: Reporting of Factorial Randomized Trials: Extension of the CONSORT 2010 Statement
JAMA. 2023;330(21):2106-2114. doi:10.1001/jama.2023.19793
Table Title:
Example of a 2 × 2 Factorial Randomized Triala A and B are factors.
b Active A and placebo A are levels within factor A and high-dose B and low-dose B are levels within factor B. Low-dose B is taken as the control condition for factor B.
c Active A plus high-dose B, active A plus low-dose B, placebo A plus high-dose B, and placebo A plus low-dose B are the treatment groups. In a “full” factorial trial all participants are eligible to be randomized between each of the 4 treatment groups; in a “partial” factorial trial, a subset of participants would only be randomized between high- and low-dose B and assigned to placebo A without randomization. In a “factorial” analysis, all participants allocated to intervention A (active A plus low-dose B and active A plus high-dose B) are compared against those not allocated to A (placebo A plus low-dose B and placebo A plus high-dose B), and similarly for the comparison for intervention B. In a “multiarm” analysis, each of the treatment groups are compared against control (eg, active A plus high-dose B, active A plus low-dose B, and placebo A plus high-dose B are all compared against placebo A plus low-dose B).
Date of download: 1/8/2025
Copyright 2023 American Medical Association. All Rights Reserved.
