# Supplementary material for: Reporting of Factorial Randomized Trials Extension of the CONSORT 2010 Statement
Source: JAMA. Author manuscript; Available in PMC 2025 Jan 25. (PMC7617336; doi:10.1001/jama.2023.19793)
Supplement: figure-Fig 2 [file EMS202149-supplement-figure-Fig_2.ppt]

## Slide 1
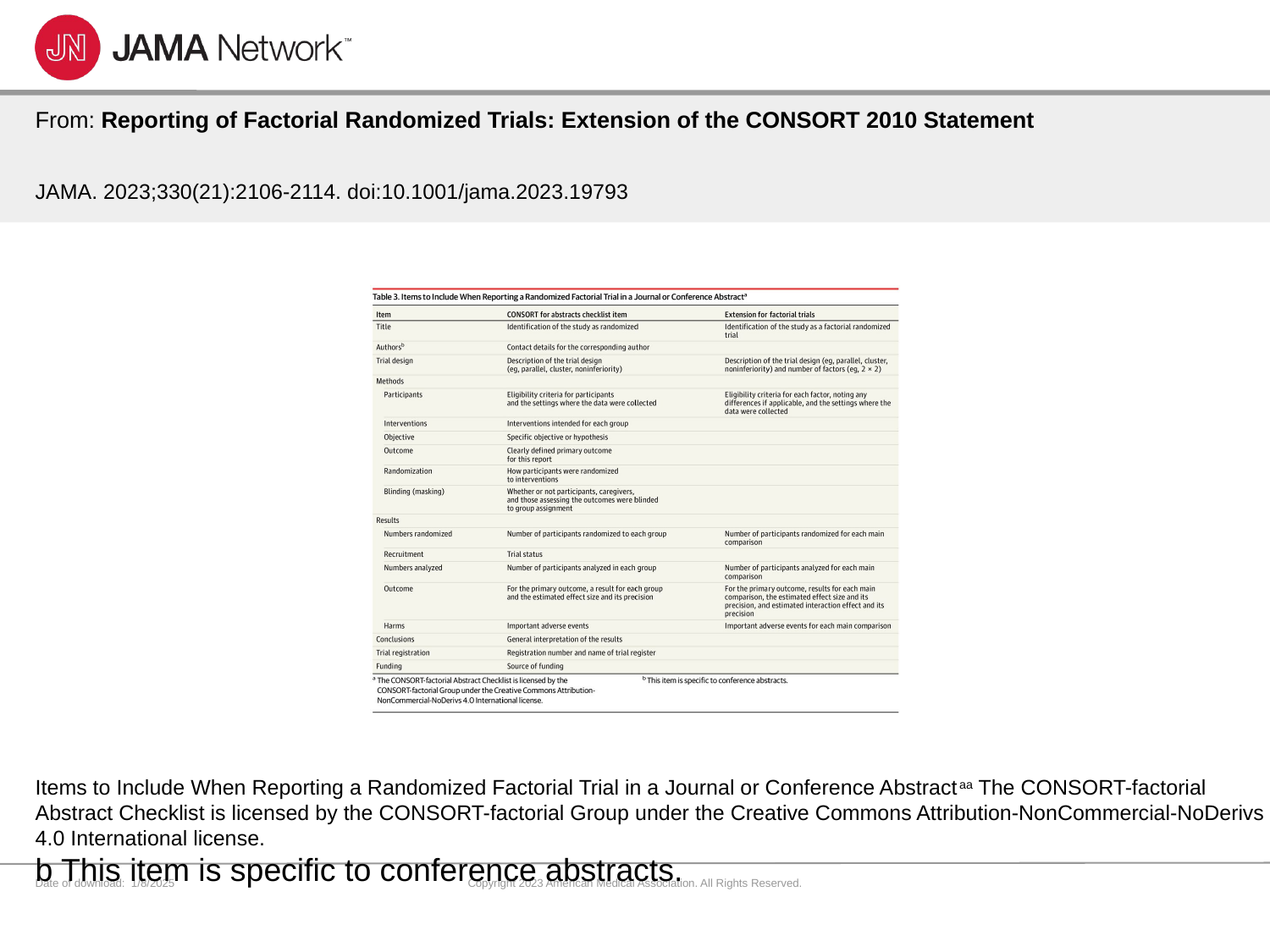

From: Reporting of Factorial Randomized Trials: Extension of the CONSORT 2010 Statement
JAMA. 2023;330(21):2106-2114. doi:10.1001/jama.2023.19793
Table Title:
Items to Include When Reporting a Randomized Factorial Trial in a Journal or Conference Abstractaa The CONSORT-factorial Abstract Checklist is licensed by the CONSORT-factorial Group under the Creative Commons Attribution-NonCommercial-NoDerivs 4.0 International license.
b This item is specific to conference abstracts.
Date of download: 1/8/2025
Copyright 2023 American Medical Association. All Rights Reserved.
