# Supplementary material for: Reporting of Factorial Randomized Trials Extension of the CONSORT 2010 Statement
Source: JAMA. Author manuscript; Available in PMC 2025 Jan 25. (PMC7617336; doi:10.1001/jama.2023.19793)
Supplement: figure-Fig 3 [file EMS202149-supplement-figure-Fig_3.ppt]

## Slide 1
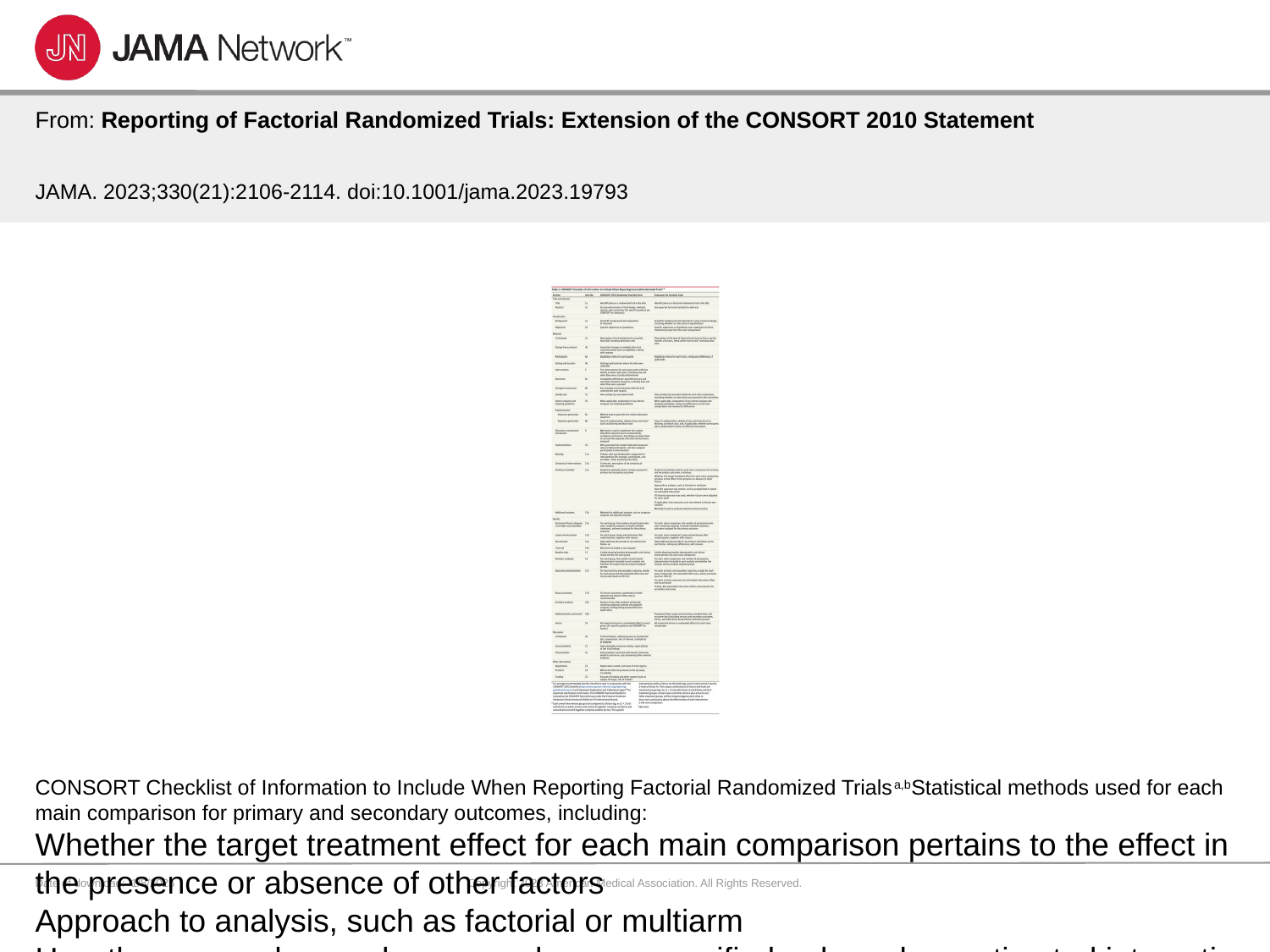

From: Reporting of Factorial Randomized Trials: Extension of the CONSORT 2010 Statement
JAMA. 2023;330(21):2106-2114. doi:10.1001/jama.2023.19793
Table Title:
CONSORT Checklist of Information to Include When Reporting Factorial Randomized Trialsa,bStatistical methods used for each main comparison for primary and secondary outcomes, including:
Whether the target treatment effect for each main comparison pertains to the effect in the presence or absence of other factors
Approach to analysis, such as factorial or multiarm
How the approach was chosen, such as prespecified or based on estimated interaction
If factorial approach was used, whether factors were adjusted for each other
If applicable, how nonconcurrent recruitment to factors was handled
Method(s) used to evaluate statistical interaction(s)
 Additional analyses12bMethods for additional analyses, such as subgroup analyses and adjusted analysesResults Participant flow (a diagram is strongly recommended)13aFor each group, the numbers of participants who were randomly assigned, received intended treatment, and were analyzed for the primary outcomeFor each main comparison, the number of participants who were randomly assigned, received intended treatment, and were analyzed for the primary outcome Losses and exclusions13bFor each group, losses and exclusions after randomization, together with reasonsFor each main comparison, losses and exclusions after randomization, together with reasons Recruitment14aDates defining the periods of recruitment and follow-upDates defining the periods of recruitment and follow-up for each factor, noting any differences, with reasons Trial end14bWhy the trial ended or was stopped Baseline data15A table showing baseline demographic and clinical characteristics for each groupA table showing baseline demographic and clinical characteristics for each main comparison Numbers analyzed16For each group, the number of participants (denominator) included in each analysis and whether the analysis was by original assigned groupsFor each main comparison, the number of participants (denominator) included in each analysis and whether the analysis was by original assigned groups Outcomes and estimation17aFor each primary and secondary outcome, results for each group and the estimated effect size and its precision (such as 95% CI)For each primary and secondary outcome, results for each main comparison, the estimated effect size, and its precision (such as 95% CI)
For each primary outcome, the estimated interaction effect and its precision
If done, the estimated interaction effects and precision for secondary outcomes
 Binary outcomes17bFor binary outcomes, presentation of both absolute and relative effect sizes is recommended Ancillary analyses18aResults of any other analyses performed, including subgroup analyses and adjusted analyses, distinguishing prespecified from exploratory Additional data summariesc18bParticipant flow, losses and exclusions, baseline data, and outcome data (including primary and secondary outcomes, harms, and adherence) presented by treatment groupsb Harms19All important harms or unintended effects in each group (for specific guidance see CONSORT for harms)All important harms or unintended effects for each main comparisonDiscussion Limitations20Trial limitations, addressing sources of potential bias, imprecision, and, if relevant, multiplicity of analyses Generalizability21Generalizability (external validity, applicability) of the trial findings Interpretation22Interpretation consistent with results, balancing benefits and harms, and considering other relevant evidenceOther information Registration23Registration number and name of trial registry Protocol24Where the full trial protocol can be accessed, if available Funding25Sources of funding and other support (such as supply of drugs), role of fundersa It is strongly recommended that this checklist is read in conjunction with the CONSORT 2010 checklist (https://www.equator-network.org/reporting-guidelines/consort/) and Statement Explanation and Elaboration paper for important clarification on the items. The CONSORT-factorial Checklist is licensed by the CONSORT-factorial Group under the Creative Commons Attribution-NonCommercial-NoDerivs 4.0 International license.
b Each overall intervention group to be compared is a factor (eg, in a 2 × 2 trial with factors A and B, active A and control A together comprise one factor and active B and control B together comprise another factor). The specific interventions within a factor are the levels (eg, active A and control A are the 2 levels of factor A). The unique combinations of factors and levels are treatment groups (eg, in a 2 × 2 trial with factors A and B there will be 4 treatment groups: active A plus control B, active A plus active B, etc). What treatment groups will be compared against each other to draw main conclusions about the effectiveness of each intervention is the main comparison.
c New item.
Date of download: 1/8/2025
Copyright 2023 American Medical Association. All Rights Reserved.
